# Membrane insertion and assembly of epitope-tagged gp9 at the tip of the M13 phage

**DOI:** 10.1186/1471-2180-11-211

**Published:** 2011-09-26

**Authors:** Martin Ploss, Andreas Kuhn

**Affiliations:** 1 Institute of Microbiology and Molecular Biology, Garbenstrasse 30, University of Hohenheim, 70599 Stuttgart, Germany

## Abstract

**Background:**

Filamentous M13 phage extrude from infected *Escherichia coli *with a tip structure composed of gp7 and gp9. This tip structure is extended by the assembly of the filament composed of the major coat protein gp8. Finally, gp3 and gp6 terminate the phage structure at the proximal end. Up to now, gp3 has been the primary tool for phage display technology. However, gp7, gp8 and gp9 could also be used for phage display and these phage particles should bind to two different or more surfaces when the modified coat proteins are combined. Therefore, we tested here if the amino-terminal end of gp9 can be modified and whether the modified portion is exposed and detectable on the M13 phage particles.

**Results:**

The amino-terminal region of gp9 was modified by inserting short sequences that encode antigenic epitopes. We show here that the modified gp9 proteins correctly integrate into the membrane using the membrane insertase YidC exposing the modified epitope into the periplasm. The proteins are then efficiently assembled onto the phage particles. Also extensions up to 36 amino acid residues at the amino-terminal end of gp9 did not interfere with membrane integration and phage assembly. The exposure of the antigenic tags on the phage was visualised with immunogold labelling by electron microscopy and verified by dot blotting with antibodies to the tags.

**Conclusions:**

Our results suggest that gp9 at the phage tip is suitable for the phage display technology. The modified gp9 can be supplied *in trans *from a plasmid and fully complements M13 phage with an amber mutation in gene 9. The modified phage tip is very well accessible to antibodies.

## Background

The bacteriophage M13 is assembled during a secretion process in the cytoplasmic membrane of *Escherichia coli*. Membrane inserted phage proteins contact the single stranded phage DNA in an helical array and pass through the outer membrane by a porin-like structure composed of gp4 [1]. In the inner membrane a protein complex probably consisting of gp1, gp11 and thioredoxin catalyses the assembly process [2]. First, membrane inserted gp7 and gp9 proteins form a tip structure [3] that is extended by a multiple array of gp8 proteins, the major coat protein. Gp8 is synthesised as a precursor protein, termed procoat, that is inserted into the inner membrane by the YidC protein [4,5] and is then processed by leader peptidase [6]. After processing, the transmembrane coat proteins assemble into oligomers and bind to the viral DNA forming the nascent phage filament [7,8]. This filament traverses the outer membrane through the gp4 complex [1]. Finally, the membrane inserted gp3 and gp6 proteins are assembled onto the extruding phage at the proximal end of the virion terminating phage assembly.

The gp3 protein has been extensively used for the phage display technology. Since gp3 is engaged in the adsorption of the phage onto the host cell certain restrictions on the infectivity of the modified phage have to be encountered [9]. This might be different for gp9 modifications since this protein is localized at the distal end of the filamentous phage particle. Previously, it has been shown that gp9 is accessible in the phage particle [3]. Therefore, gp9 might be a good target for phage display technology [10]. In addition, an attractive idea is to have both ends supplied with functional peptide moieties applicable as molecular measures or bifunctional binders.

Gp9 is a 32 amino acid long protein that is synthesised without a signal sequence. It is thought that the membrane-inserted protein displays its N-terminus into the periplasm. However, the first amino-terminal 17 residues are hydrophobic and it is questionable whether the protein spans the entire bilayer. One possibility to explore this is to fuse hydrophilic peptides onto the N-terminus. When these modified gp9 proteins are inserted into the membrane their amino-terminal region can be analysed whether they are exposed in the periplasm. Therefore, we have fused short antigenic peptides to the N-terminus of gp9 between the residues 2 and 3. They extend the protein by 17 to 36 amino acid residues. The proteins are inserted into the membrane and efficiently assemble onto phage progeny particles since they can substitute for the wild-type protein. Also, the antigenic epitopes are detectable with gold-labelled antibodies by electron microscopy.

## Results

### Antigenic epitopes at the N-terminus of M13 gp9

**Figure 1:**
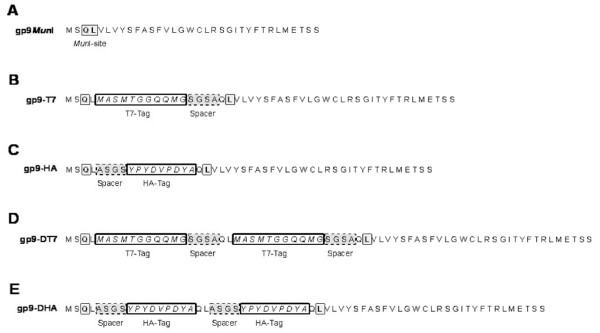
**Variants of M13 gp9 proteins**. Schematic overview of the gp9 variants used in this work. Into the wild-type a *Mun*I restriction site was introduced between codon 2 and 3 resulting in two additional residues in gp9*Mun*I (A). Into this *Mun*I site short sequences were introduced encoding for the T7 tag in gp9-T7 (B) and for the HA tag in gp9-HA (C). In addition, a double tag was introduced into gp9 generating gp9-DT7 (D) and gp9-DHA (E), respectively. The protein sequence of each mutant is given in the single letter code.

**Figure 2:**
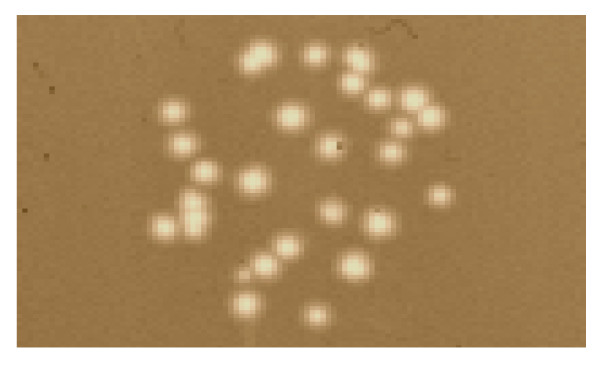
**Plaque formation of M13am9 with gp9-HA coat protein**. *E. coli *K38 bearing pMS-g9-HA was mixed with LB top agar containing 1 mM IPTG and poured on an agar plate. After solidification, M13*am*9 phage was applied and incubated at 37 °C overnight.

**Figure 3:**
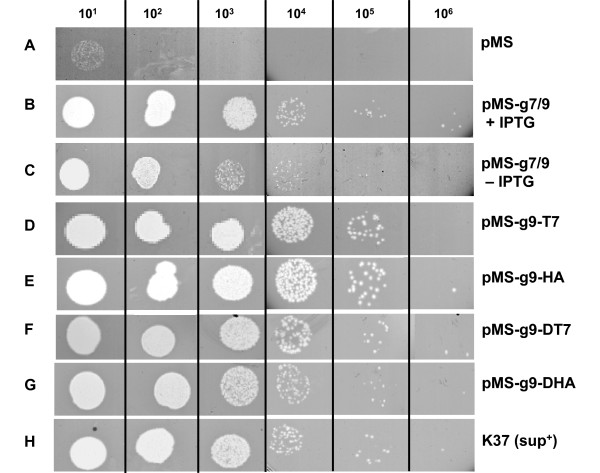
**Complementation of M13am9 infections by plasmid-expressed gp9**. *E. coli *K38 bearing the respective plasmid was mixed with LB top agar containing 1 mM IPTG and poured on an agar plate. After solidification, 10 μL drops of serial diluted M13*am*9 phage suspensions were applied. The bacterial lawn with K38 cells bearing the empty plasmid only allowed the plaque formation of revertant phage (A). K38 cells expressing the wild-type gp9 from the plasmid (B) showed plaque formation at the 10^5^-fold dilution, similar to the suppressor cells K37 (H). When no IPTG was added to the plate plaque formation was reduced (C). Cells expressing the modified gp9 proteins all showed efficient plaque formation. Gp9-T7 (D), gp9-HA (E), gp9-DT7 (F) and gp9-DHA (G) were analysed.

To study the assembly of M13 gp9, genetic variants were constructed that extend the N-terminal region of the protein with antigenic epitopes. Two antigenic peptides, a 17 amino acid residues long T7-tag and a 15 residues long HA-tag were fused to the N-terminus of gp9, respectively (Figure 1). This was performed on a gene 9 copy on a pMS119 plasmid using a unique *Mun*I restriction site that was engineered between the codons 2 and 3 to generate gp9*Mun*I. Into this site, DNA fragments encoding the tag sequences were introduced. In addition, longer fragments were introduced which encode two copies of the antigenic tag sequences, resulting in additional 36 and 32 residues in pMS-g9-DT7 and pMS-g9-DHA, respectively. Then, the functionality of the modified proteins was tested by complementation of an M13*am9 *phage infection (Figure 2 and 3). *E. coli *K38 bearing the corresponding plasmid was grown overnight in LB medium and plated with top agar containing 1 mM IPTG. After solidification of the top agar 10 μL of a phage suspension was applied on top of the agar. Plaque formation was observed after incubation at 37°C overnight. When the cells with pMS-g9-HA were infected with M13*am*9 clear plaques with a turbid zone were visible on the bacterial lawn (Figure 2). Whereas no plaques appeared with the K38 cells containing the pMS plasmid (Figure 3, panel A), pMS-g7/9 transformed cells showed plaque formation down to the 10^5^-fold dilution step (panel B). In the absence of IPTG (panel C) plaque formation was observed at the 10^4^-fold dilution which is most likely due to a low expression or to recombination events. When K38 cells with the pMS-g9-T7 (panel D) or with pMS-g9-HA (panel E) were used plaque formation was evident down to the 10^5^-fold dilution step. Similarly, the plasmids encoding the double tags (panels F and G) showed efficient plaque formation, as it was observed on the plates with the suppressor containing *E. coli *K37 cells (panel H). These results suggest that the gp9 variants expressing the epitope-tagged proteins are functional and allow normal phage propagation.

### Expression of the modified gp9 protein in E. coli

**Figure 4:**
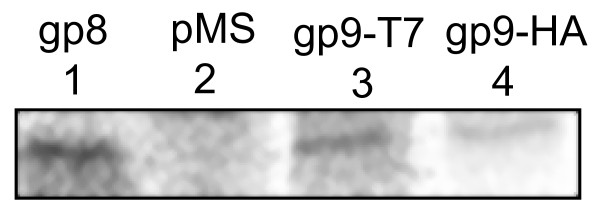
**Expression of gp9-T7 from a plasmid**. Exponentially growing *E. coli *K38 cells bearing a plasmid encoding M13 gp8 (lane 1), the empty pMS plasmid (lane 2), pMS-g9-T7 (lane 3) and pMS-g9-HA (lane 4), respectively, were induced for 10 min with IPTG and pulse-labelled with ^35^S-methionine for 10 min. The proteins were precipitated with trichloroacetic acid (TCA) and immunoprecipitated with antiserum to gp8 (lane 1), to T7 (lane 2, 3) and to HA (lane 4), respectively. SDS tricine PAGE was used to separate the proteins and the radioactivity was visualised by phosphorimaging.

The plasmid-encoded gp9 variants were analysed for expression in *E. coli *K38. The cells were grown at 37°C to the early exponential phase in M9 minimal medium. Protein expression was induced by adding 1 mM IPTG and after 10 min the newly synthesised proteins were pulse-labelled for 10 min with ^35^S-methionine. The total bacterial proteins were TCA precipitated to remove the non-incorporated ^35^S-methionine and immunoprecipitated using an antiserum to the T7 tag or to the HA tag, respectively (Figure 4). Since gp9 is a very small protein of 32 amino acids containing only two methionines the protein band on a SDS tricine PAGE is difficult to visualise. When comparing the protein pattern of cells expressing gp9-T7 (lane 3) with cells containing the empty plasmid (lane 2) a protein band of about 5.5 kDa was observed. Also a weak band of gp9-HA (lane 4) was visible on the gel. The size of the protein was estimated in relation of the major coat protein gp8 shown in lane 1. Since the 50 amino acid residues long gp8 has a molecular weight of 5.2 kDa, the gp9-T7 with 51 residues and gp9-HA with 49 residues are proteins of very similar molecular weight.

### Membrane insertion of gp9-T7

**Figure 5:**
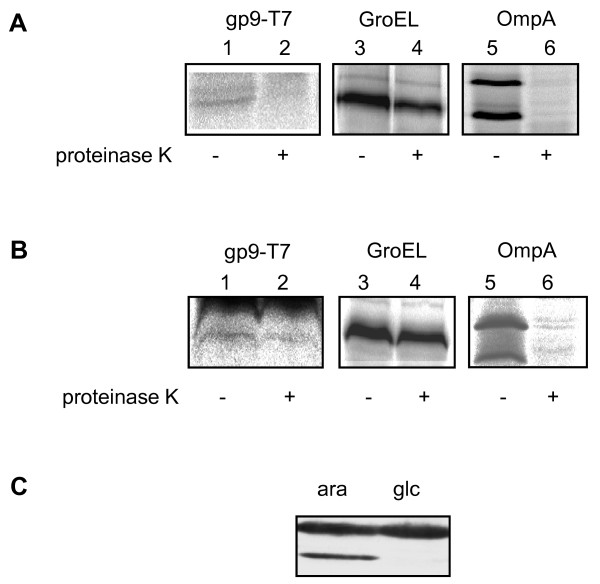
**Membrane insertion of gp9-T7 in E. coli K38 and JS7131**. Exponentially growing *E. coli *K38 cells (panel A) and JS7131 (panel B), respectively, containing the plasmid pMSg9-T7 were pulse-labelled with ^35^S-methionine for 10 min. The cells were converted to spheroplasts and incubated on ice for 1 h either in the presence or absence of 0.5 mg/mL proteinase K. The samples were immunoprecipitated with antiserum to T7 (lanes 1, 2), to GroEL (lanes 3, 4) and to OmpA (lanes 5, 6), respectively, and analysed on SDS PAGE and phosphorimaging. (C) The depletion of YidC in the JS7131 cells grown in M9 medium with 0.2% glucose (glc) was verified by Western blot using an antibody to YidC. As control for the non-depleted conditions, the JS7131 cells were grown in the presence of 0.2% arabinose (ara).

The membrane insertion of gp9 with the N-terminal T7 tag was analysed in *E. coli *K38 cells bearing the pMS-g9-T7 plasmid. The gp9-T7 protein was expressed as described above. The cells were converted to spheroplasts and analysed by protease mapping (Figure 5A). The protein immunoprecipitated with antiserum to the T7 tag was readily digested by proteinase K added to the outside of the spheroplasts (lane 2). This suggests that the antigenic tag of gp9 was accessible to the protease at the periplasmic surface, whereas the cytoplasmic GroEL protein was protected from digestion (lane 4). Further, the periplasmic portion of the OmpA protein was digested by the proteinase K (lane 6) confirming the proteolytic activity.

The insertion of gp9-T7 into the membrane was then investigated in *E. coli *JS7131. In these cells, the membrane insertase YidC can be depleted when the cells are grown in the presence of glucose [4]. After 2 h growth under glucose conditions the cells were pulse-labelled with ^35^S-methionine for 10 min and converted to spheroplasts. The protease mapping (Figure 5B) shows that the YidC depleted cells did not allow the digestion of the T7-epitope at the N-terminus of gp9 (lane 2). These results suggest that the membrane insertion of gp9-T7 is YidC-dependent. In both cases, the integrity of the spheroplasts was verified by the protection of GroEL (lane 4) and the proteolytic activity was corroborated by the accessibility of the OmpA protein (lane 6).

### Assembly of gp9 variant proteins onto phage

**Figure 6:**
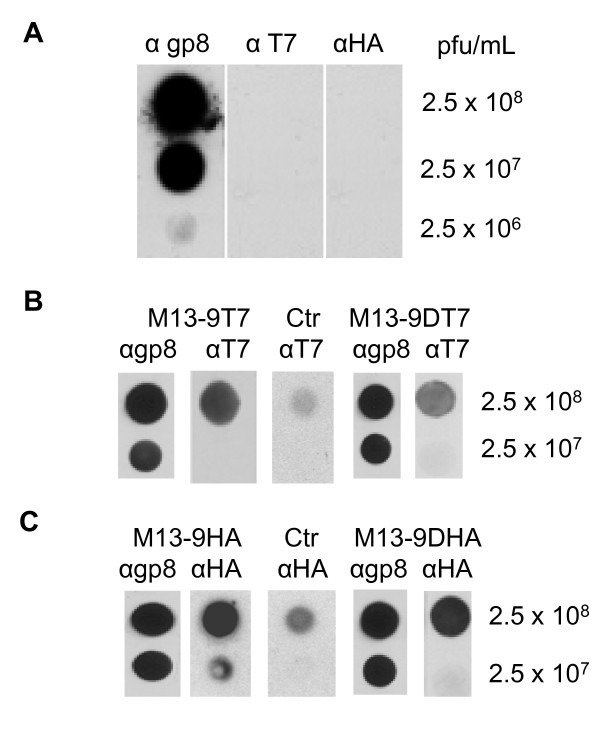
**Presentation of the antigenic tags on gp9 of phage particles**. (A) M13 phage (panel A) was applied onto nitrocellulose membrane and incubated with antibody to gp8, T7 tag and HA tag, respectively, at the indicated concentrations. To visualise the phage a peroxidase-linked secondary antibody was applied and analysed by chemoluminescence. (B) M13*am*9 phage grown in *E. coli *K38 cells bearing a plasmid encoding gp9-T7 or gp9-DT7, respectively, were incubated with antibody to T7 and treated as described above. (C) M13*am*9 phage propagated in *E. coli *K38 cells bearing a plasmid encoding gp9-HA or gp9-DHA, respectively, were incubated with antibody to HA and treated as described above. For controls (Ctr), uninfected cultures were tested under identical conditions.

Assembly of the plasmid-encoded variants onto phage was first followed by dot-blot analysis of phage particles. M13*am*9 infections in *E. coli *K38 bearing a plasmid coding for one of the gp9 variants were performed and the progeny phage were collected and titrated. Equal amounts of phage was applied on nitrocellulose, incubated with antiserum to M13 gp8, to T7 tag or to the HA tag, respectively. The reaction with a secondary peroxidase coupled antibody was analysed by chemoluminescence (Figure 6). Whereas the infecting M13*am*9 phage reacted only to the anti gp8 serum (panel A), the phage grown in cells with pMS-g9-T7 clearly reacted with the T7 serum (panel B). Similarly, phage from cells expressing the double tag gp9-DT7 also reacted with the serum to the T7 tag. Strong signals were obtained with gp9 proteins with the HA epitopes (panel C) whereas the uninfected K38 cells expressing gp9-T7 or gp9-HA showed only a low signal in the corresponding supernatants. This verifies that the plasmid encoded gp9 proteins with the epitope tags were efficiently assembled onto the phage particles.

**Figure 7:**
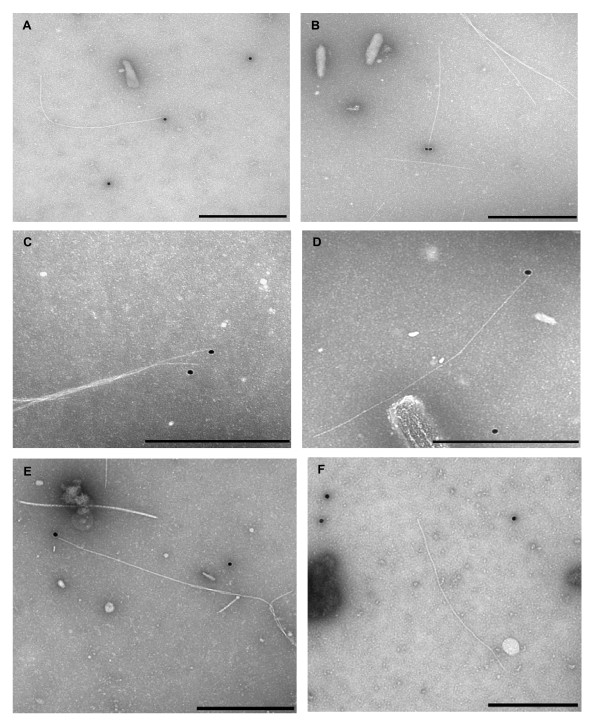
**Binding of conjugated gold to M13 phage with modified gp9**. M13*am9 *phage propagated in *E. coli *K38 cells bearing a plasmid encoding gp9-DT7 or gp9-DHA, respectively, was tested for the presentation of the tag at the tip of the phage particles. The phage was incubated with the respective antibody and to protein A coupled immunogold particles (20 nm). M13*am*9-gp9-DT7 phage (A, B) and M13*am*9-gp9-DHA phage (C - E) were applied onto carbon coated grids, stained with 5% phosphotungstic acid and analysed by electron microscopy. For a control, M13 phage was applied (F). The scale bars correspond to 500 nm.

The exposure of the antigenic epitopes on the phage particles was then tested with immunogold (Figure 7). First, phage was incubated with the respective serum, then with protein A coupled immunogold particles (20 nm) and applied to coated copper grids. After staining with 5% phosphotungstic acid (pH 7) the phage particles were inspected. Several gold nanoparticles were bound to the tip of individual phages either with the T7 tag (panel A) the double T7 tag (panel B), or the double HA tag (panel C, D, E). The parental M13*am*9 phage, used as a control showed no binding of the gold nanoparticles to the tip (panel F). In contrast, for both complemented phage particles we found that about 30% of the gold nanoparticles were bound to phage particles and about 20% of the phage had a gold nanoparticle bound at the tip. Taken together, the analysis shows that the modified gp9 proteins are well exposed and accessible to antibodies.

## Discussion

The minor coat protein gp9 of the filamentous phage M13 is exposed from the phage particle and can be modified with short peptides. Here we have shown that peptides of 17, 18, 32 and 36 amino acid residues can be incorporated into the amino-terminal region of the protein without interfering with membrane insertion and assembly of the phage. The epitopes of these peptides are accessible by antibodies and allow binding of gold nanoparticles that can be visualised by electron microscopy. This implicates that gp9 could be used for the phage display methodology allowing a combination with the well-established display of modified gp3. Previous experiments have shown that gp9 of the closely related fd phage is localised at the distal end, together with gp7 [3]. In that study, it was also shown, that the gp9 protein is exposed to the surface in contrast to gp7. However, a fusion with glutathione-S-transferase (GST) did not support phage production suggesting that membrane insertion or phage assembly is inhibited. Since GST is a folded structure of about 35 kDa we tested smaller fusion proteins that may be tolerated for membrane insertion and phage assembly. By introducing short antigenic sequences between the amino acid residues 2 and 3 of gp9 on a plasmid membrane insertion and phage assembly was followed. Also, longer fusions consisting of 32 and 36 additional residues that code for two tandem tags were constructed. Intriguingly, all gp9 fusion proteins complement an amber-9 phage infection and lead to progeny production up to wild-type levels. When the phage progeny particles were analysed for the presentation of their antigenic epitopes we observed by dot-blot analysis (Figure 6) and immunogold labelling (Figure 7) a clearly positive response. We conclude that the amino-terminal end of gp9 is capable to accept modifications and provides a new possibility for phage display.

The extended amino-terminal region with an antigenic tag allowed the investigation of the membrane insertion of gp9 in detail. Previously, it had been shown by FTIR spectroscopy that the membrane-inserted protein has a high α-helical conformation and adopts a transmembrane conformation [11]. In a short pulse, the synthesised gp9 was radioactively labelled and analysed for membrane insertion by protease added to the outside of the membrane (Figure 5). Indeed, the protease removed the antigenic tag at the N-terminus of gp9, whereas the cytoplasmic GroEL protein was protected from proteolysis. When the same experiment was performed in cells that were depleted for YidC, gp9 was not digested suggesting that it was not inserted into the membrane under these conditions. We conclude, that gp9 uses the YidC-only pathway for insertion similar to gp8 [4,5]. In contrast to our *in vivo *experiments, earlier *in vitro *data with artificial liposomes consisting of DOPC and DOPG had suggested that the gp9 protein inserts sponanteously into the membrane [12].

Very recently, similar gp9 variants to our gp9 fusion proteins were described that allowed a display on the phage [10]. In contrast to our work, a phagemid system was used and the N-terminus of gp9 fusion protein had a *pelB *signal sequence attached. This likely changes the route of membrane insertion to the Sec-translocase and allows the translocation of large N-terminal domains across the cytoplasmic membrane. Compared to the phagemid system used in previous reports [10,13-15], we present a new method of gp9 phage display which allows a polyvalent phage display without the need of an N-terminal signal sequence and helper phage infection. In our system the only gp9 copy available is the modified gp9 protein on a plasmid when amber 9 phage was used. Therefore, all gp9 proteins on the phage particle possess the modified N-terminus. Further, our system allows to clearly determine the extend of interference of the modified protein with the propagation cycle of the phage. When no interference is observed and the gp9 fusion protein can substitute for the wild-type, amber 9 phage can be used to generate modified phage particles without a modification of the phage genome. This application might be useful for systems that are sensitive to genetically modified organisms according to (GMO)-rules.

## Conclusions

Bacteriophage M13 is suitable for phage display not only with a modified gp3 but also with a modified gp9 which is a minor coat protein at the phage tip. The modified gp9 protein can be supplied *in trans *from a plasmid and fully complements an amber 9 phage mutant. The modified phage tip is very well accessible to specific antibodies.

## Methods

### Phage, plasmid and bacterial strains

M13 phage was from our lab collection [16]. M13*am*9 with an amber mutation in the second codon of gIX was constructed by site-directed mutagenesis [17]. For the construction of gp9-T7, gp9-DT7, gp9-HA and gp9-DHA RF-DNA of M13*mp*19 served as template for PCR amplification. The PCR amplified gIX was subcloned into pMS119 [18] and an unique *Mun*I restriction site was introduced by QuikChange^TM^* in vitro *mutagenesis between the codons 2 and 3. Into this site RF-DNA of M13*mp*19 served as template for the amplification of gIX by PCR. The gIX fragment was subcloned into pMS119, DNA fragments encoding the T7 and HA tag sequences were introduced by ligation, resulting in pMS-g9-T7 and pMS-g9-HA. Also, longer epitopes were introduced to construct pMS-g9-DT7 and pMS-g9-DHA, respectively. For protein expression and complementation experiments *E. coli *K38 (HfrC *T2R rel*A1 *pit*-10 *spo*T1 *ton*A22 *omp*F627 *pho*A4 λ^-^) [19] was transformed as a non-suppressor strain. *E. coli *K37 (HfrC *sup*D32 *rel*A1 *pit*-10 *spo*T1 *ton*A22 *omp*F627 *pho*A4 *T2R *λ^-^) [19,20] was used as a suppressor strain and *E. coli *JS7131 (MC1060 Δ*yidC attB*::*R6Kori ParaBADyidC*^+ ^Spec^r^) as a depletion strain of the membrane insertase YidC [4].

### Complementation test of phage expressing modified gp9 proteins

On agar plates 4 mL melted LB top agar (47°C) containing 1 mM IPTG was mixed with 500 μL of a fresh *E. coli *K38 overnight culture bearing either pMS-g9/7 pMS-g9-T7, pMS-g9-DT7, pMS-g9-HA or pMS-g9-DHA. After solidification of the top agar, 10 μL of a phage suspension was applied on top of the agar from serial dilutions of a phage stock. Plaque formation was observed after incubation at 37°C overnight.

### Expression of the modified gp9 proteins

2 mL cultures of *E. coli *K38 bearing plasmids encoding a respective gp9 variant were grown at 37°C to the early exponential phase in M9 minimal medium. Protein expression was induced by adding 1 mM IPTG and 10 min later the newly synthesised proteins were pulse-labelled for 10 min with 20 μCi ^35^S-methionine. To remove the non-incorporated ^35^S-methionine the total bacterial proteins were precipitated with 12% TCA on ice overnight, washed with cold acetone and resuspended in 10 mM Tris/HCl 2% SDS, pH 8.0. The samples were immunoprecipitated with antiserum to the T7 tag (Novagen) or to the HA tag (Roche), respectively, and analysed by SDS tricine PAGE and phosphorimaging.

### Membrane insertion of gp9

To test the membrane insertion of gp9, *E. coli *K38 bearing pMS-g9-T7 was grown to the early exponential phase in M9 minimal medium. Cells were induced for 10 min with 1 mM IPTG and labelled with ^35^S-methionine for 10 min. To generate spheroplasts, the cells were centrifuged at 12 000 g for 3 min and resuspended in 500 μL of ice-cold spheroplast buffer (40% w/v sucrose, 33 mM Tris/HCl, pH 8.0). Lysozyme (5 μg/mL, final concentration) and 1 mM EDTA were added for 15 min. Aliquots of the spheroplast suspension were incubated on ice for 1 h either in the presence or absence of 0.5 mg/mL proteinase K. The samples were precipitated with 12% TCA, washed with cold acetone and resuspended in 10 mM Tris/HCl, 2% SDS, pH 8.0 and immunoprecipitated with antibodies against T7, OmpA (a periplasmic control), or GroEL (a cytoplasmic control). Samples were analysed by SDS tricine PAGE and phosphorimaging.

### In vivo assay of YidC dependent membrane insertion

To test the requirement of YidC for the membrane insertion of gp9-T7, the YidC depletion strain *E. coli *JS7131 bearing pMS-g9-T7 was grown to the early exponential phase in LB with 0.2% arabinose. After back-dilution, the cells were grown in M9 minimal medium with either 0.2% arabinose (YidC^+^) or 0.2% glucose (YidC^-^) for 2 h. To induce expression of gp9-T7, 1 mM IPTG was added and after 10 min the cells were pulse-labelled with ^35^S-methionine for 10 min and then converted to spheroplasts by lysozyme treatment as described above. Samples were immunoprecipitated with antibodies to T7, OmpA (a periplasmic control), or GroEL (a cytoplasmic control). For testing the YidC depletion, samples of the cultures were drawn and precipitated with TCA (12%, final concentration), washed with cold acetone, resuspended in 10 mM Tris/HCl, 2% SDS, pH 8.0 and analysed by SDS/PAGE and Western blot using YidC antiserum.

### M13*am*9 phage presenting gp9 variant proteins

50 mL cultures of *E. coli *K38 cells harbouring either pMSg9-T7, pMSg9-DT7, pMSg9-HA or pMSg9-DHA were grown at 37°C in LB-medium to a density of 2 × 10^8 ^cells/mL. The expression of the gp9 variant proteins was induced by adding 1 mM IPTG and the cells were infected with M13*am*9 at m.o.i 10. Adsorption of the phage was allowed for 5 min at room temperature without shaking. Subsequently, the infected cells were shaken overnight at 37°C. The phage was harvested from the supernatant after removing the cells by centrifugation. Then, the phage titer was determined by serial dilutions on *E. coli *K37. Every dilution was plated three times on LB agar plates to control variations in plating and pipetting. The agar plates were incubated at 37°C overnight and the plaques were counted and averaged for each dilution step.

### Dot-blot analysis

For detection of the plasmid-encoded variants on the phage via dot-blot, serial dilutions of the above described phage stocks were prepared resulting in equal amounts of phage particles/400 μL for every variant. 400 μL of each suspension was adsorbed on a nitrocellulose membrane (Hybond ECL Nitrocellulose, Amersham) via dot-blot equipment (MiniFold^®^, Schleicher & Schuell) and treated overnight with blocking solution (1x Tris-buffered saline (TBS) pH 8, 5% non-fat dry milk w/v). The blot was washed three times with 1x TBS and incubated with antiserum to M13 gp8, to T7 or to HA tag, respectively. The presence of gp9 variants was analysed with a secondary peroxidase-coupled antibody by chemoluminescence.

### Immunogold labelling of M13gp9 variant phage for TEM

For testing the exposure of an antigenic epitope 50 μL of each phage stock solution (about 10^11 ^phage/mL) of M13gp9-DT7 and M13gp9-DHA was incubated with 1 × TBS containing 0.1% BSA for 30 min to avoid unspecific binding of the primary antibody to the sample. Each sample was then incubated with the respective serum (diluted 1:20 in 1x TBS) for 1 h. Then, protein A coupled immunogold particles (Protein A - 20 nm colloidal gold, Sigma-Aldrich) was added 1:20 in 1x TBS for 1 h. After immunogold labelling, 10 μL of the phage stock solution was adsorbed on carbon-coated copper grids (Athene 200, Plano, Wetzlar/Germany) that had been glow discharged shortly before use [21]. The suspensions were allowed to adsorb for 5 min, unbound material was removed by touching the grid to filter paper. The grid was then washed by touching the surface of a drop of distilled water for 2 sec. The excess water was removed by touching the grid to filter paper. A drop (5 μL) of 5% phosphotungstic acid (pH 7) was then applied to the grid and after 30 sec the excess stain was removed by touching the grid to a drop (50 μL) of ddH_2_0 for 2 sec. The excess liquid was drawn off with filter paper. The grid was dried at room temperature and examined by electron microscopy.

## Authors' contributions

MP carried out all experiments. AK designed the project and wrote the manuscript. Both authors read and approved the final manuscript.
