# Erratum to: Membrane insertion and assembly of epitope-tagged gp9 at the tip of the M13 phage

**DOI:** 10.1186/s12866-017-1072-9

**Published:** 2017-08-21

**Authors:** Martin Ploss, Andreas Kuhn

**Affiliations:** Institute of Microbiology and Molecular Biology, Garbenstrasse 30, University of Hohenheim, 70599 Stuttgart, Germany

## Erratum

After publication of our article [1], we became aware that during the assembly of panel A in Fig. 6 a wrong control lane (α T7) had been included. The results and conclusions are not affected by this error.

The corrected Fig. 6 is shown as follows:

**Fig. 6:**
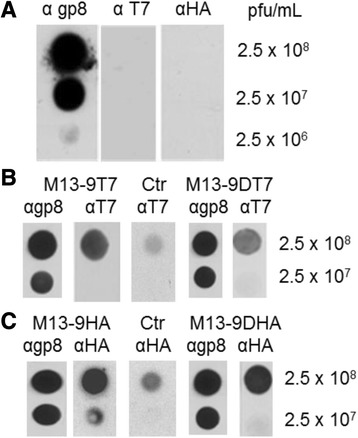
Presentation of the antigenic tags on gp9 of phage particles. (**a**) M13 phage (panel **a**) was applied onto nitrocellulose membrane and incubated with antibody to gp8, T7tag and HA tag, respectively, at the indicated concentrations. To visualize the phage a peroxidase-linked secondary antibody was applied and analysed by chemoluminescence. **b** M13 *am*9 phage grown in *E. coli* K38 cells bearing a plasmid encoding gp9-T7 or gp9DT7, respectively, were incubated with antibody to T7 and treated as described above. **c** M13 *am*9 phage propagated in *E. coli* K38 cells bearing a plasmid encoding gp9-HA or gp9DHA, respectively, were incubated with antibody to HA and treated as described above. For controls (Ctr), uninfected cultures were tested under identical conditions.
